# Distal Renal Tubules Are Deficient in Aggresome Formation and Autophagy upon Aldosterone Administration

**DOI:** 10.1371/journal.pone.0101258

**Published:** 2014-07-07

**Authors:** Muhammad Umar Cheema, Helle Hasager Damkier, Jakob Nielsen, Ebbe Toftgaard Poulsen, Jan J. Enghild, Robert A. Fenton, Jeppe Praetorius

**Affiliations:** 1 Department of Biomedicine, Membranes Center & InterPrET Pilot Center, Health, Aarhus University, Aarhus, Denmark; 2 Department of Molecular Biology and Genetics, iNano, Science and Technology, Aarhus University, Aarhus, Denmark; Emory University, United States of America

## Abstract

Prolonged elevations of plasma aldosterone levels are associated with renal pathogenesis. We hypothesized that renal distress could be imposed by an augmented aldosterone-induced protein turnover challenging cellular protein degradation systems of the renal tubular cells. Cellular accumulation of specific protein aggregates in rat kidneys was assessed after 7 days of aldosterone administration. Aldosterone induced intracellular accumulation of 60 s ribosomal protein L22 in protein aggregates, specifically in the distal convoluted tubules. The mineralocorticoid receptor inhibitor spironolactone abolished aldosterone-induced accumulation of these aggregates. The aldosterone-induced protein aggregates also contained proteasome 20 s subunits. The partial de-ubiquitinase ataxin-3 was not localized to the distal renal tubule protein aggregates, and the aggregates only modestly colocalized with aggresome transfer proteins dynactin p62 and histone deacetylase 6. Intracellular protein aggregation in distal renal tubules did not lead to development of classical juxta-nuclear aggresomes or to autophagosome formation. Finally, aldosterone treatment induced foci in renal cortex of epithelial vimentin expression and a loss of E-cadherin expression, as signs of cellular stress. The cellular changes occurred within high, but physiological aldosterone concentrations. We conclude that aldosterone induces protein accumulation in distal renal tubules; these aggregates are not cleared by autophagy that may lead to early renal tubular damage.

## Introduction

Aldosterone, a steroid hormone with pronounced mineralocorticoid action, is expressed specifically in terrestrial mammals to conserve Na^+^ and control body fluid volume [1], [2]. Aldosterone exerts many of its effects via a genomic pathway. Following aldosterone binding to the mineralocorticoid receptor (MR) in the cytoplasm, the aldosterone-receptor complex translocates to the nucleus and induces target gene transcription [3]. The kidney is a major site for regulating Na^+^ excretion where the hormone regulated fine tuning of the excretion occurs in the distal tubules and collecting ducts. Distal tubules are sub-divided into thick ascending limbs (TAL) and distal convoluted tubules (DCT), which empty into the collecting ducts (CD) through the connecting tubules (CNT). The epithelial cells displays aldosterone sensitivity in the late part of DCT (DCT2), the CNT, and the CD [2]. Apart from increasing abundance and/or activity of plasma membrane cation transporters such as the epithelial Na^+^ channel (ENaC), aldosterone increases the metabolic capacity of its target cells to meet the increased demand of the augmented ion transport rate [4], [5].

In general, sustained induction of gene transcription and subsequent translation also increases the demand for efficient cellular protein breakdown [6]. Both cytosolic proteins and misfolded or unprocessed membrane proteins are degraded in cytosolic proteasomes after polyubiquitination. The misfolded or unprocessed membrane proteins escape the endoplasmatic reticulum (ER) by ER associated degredation (ERAD), which eventually leads to proteasomal degredation of the protein. In order to secure cell survival, the capacity for such degradation can be increased in the unfolded protein response (UPR). However, if this mechanism is saturated, the UPR initiates apoptosis [7]. In cases where the capacity of proteosomal degradation is exceeded, proteins destined for breakdown accumulate in aggregates that, after partial de-ubiquitination by ataxin-3 [8], are transported to protein structures near the microtubule-organizing center called aggresomes [9]. The proteins accumulated in aggresomes are cleared by autophagosomes and broken down by lysosomes [10]. The accumulation of protein aggregates is a central event in the pathogenesis of several diseases. Some of these conditions affect the central nervous system, such as Alzheimers, Parkinson's, and Huntington's diseases and amyotrophic lateral sclerosis, while others cause epithelial dysfunction as in the cases of cystic fibrosis and alcoholic liver disease [11]–[13].

Chronically elevated plasma aldosterone levels can cause MR dependent renal damage at several levels [14]. Most attention has been given to the glomerular injury that leads to increased filtration of proteins and glomerulosclerosis; both of which impair renal function [15]. By contrast, few investigations have focused on the inappropriate changes in the renal tubular system, such as the proximal and distal tubular damage, which can precede micro-albuminuria and glomerular damage in diabetes mellitus [16], [17].

Based on previous unexplained observations of tubular cell inclusions upon hormone treatment, we hypothesized that the increased demand for protein synthesis in distal renal tubular cells during elevated plasma aldosterone levels exerts a pressure on the protein degradation system. Our data indicate that, 1) the proteasomal breakdown of proteins in these cells is overwhelmed during aldosterone administration and, 2) the cells are incapable of forming aggresomes and autophagomes to clear the resulting aggregates upon aldosterone administration. Furthermore, the renal cortex contained foci with early signs of epithelial stress. The current study exemplifies a condition, where the established system for cellular degradation of cytosolic proteins is not activated when required in a specific cell type *in vivo* and that there are potentially damaging consequences of protein aggregation for these cells and thereby the organ.

## Materials and Methods

### Animal experiments

Male Wistar Rats (Taconic) were given 50 µg/kg body wt/day aldosterone or vehicle for 7 days through subcutaneous osmotic minipumps (Alzet osmotic minipumps, Cupertino, CA). Rats were anesthetized with isofluorane, blood sampled and the right kidney removed and processed for immunoblotting. The left kidney was fixed by perfusion with 3% paraformaldehyde in 0.1 M PBS and post-fixed in the same buffer for 1 hour. The animal experiments were performed according to the license issued by the The Animal Experiments Inspectorate, Ministry of Food, Agriculture and Fisheries - Danish Veterinary and Food Administration. The aldosterone concentration of blood plasma samples was assessed by radioimmunoassay using Coat-a-Count Aldosterone Kit (Siemens).

### Antibodies

The primary antibodies utilized are described in Table 1. Secondary antibodies were horseradish peroxidase conjugated goat anti-rabbit IgG or donkey anti-Goat IgG (Dako). For fluorescence detection, donkey anti-rabbit –goat or -mouse Alexa Fluor 488, 555 or 633 (Invitrogen) were used. For electron microscopy, goat anti-rabbit gold particle conjugated antibodies (5 or 10 nm diameter, BioCell Research Laboratories) were applied.

**Table 1:** Primary antibodies used in the study.

Target	Abbreviation	Source	Host
Epithelial Na^+^ channel α-subunit	αENaC	Johannes Loffing	Rb
60 s ribosomal subunit L22	RPL22	Acris (AP23832PU-N)	Gt
RPL22 (Intended αENaC)	H-95	Santa Cruz (sc-21012)	Rb
Na^+^, K^+^, 2Cl^−^ cotransproter 2	NKCC2	Mark Knepper	Rb
Na^+^, Cl^−^ cotransproter	NCC	Mark Knepper	Rb
Calbindin-D28K	Calbindin	Fitzgerald (10R-C106a)	Mo
Aquaporin-2	AQP2	Lofstrand (to JP: H7661)	Rb
E-cadherin	E-cad	BD Biosciences (610181)	Mo
Proteasome marker	Proteasome 20 s	Abcam (Ab3325)	Rb
Aggresome marker	HDAC6	Santa Cruz (sc-5258)	Gt
V1-ATPase B1 subunit	H^+^-ATPase	BM Christensen [33]	Rb
Autophagosome marker	LC3	Abcam (Ab58610)	Rb
Recycling endosome	Rab11b	BD Biosciences (610656)	Ch
Early endosome marker	EEA1	BD Biosciences (610457)	Mo
Late endo-/lysosome marker	CathepsinD	R&D Systems (AF1029)	Gt
Aggresome marker	Dynactin p62	Santa Cruz (sc-55603)	Mo
Vimentin	Vimentin	Millipore (MAB3400)	Mo

Rb  =  rabbit; Mo  =  mouse; Gt  =  goat; Ch  =  chicken.

### Immunoblotting

The kidneys were homogenized (Ultra-Turrax T8 homogenizer) in ice-cold dissection buffer containing 300 mM sucrose, 25 mM imidazole, 1 mM EDTA, 8.5 µM leupeptin, and 1 mM phenylmethylsulfonyl fluoride, with pH 7.4. After centrifugation at 4,000 g for 15 min at 4°C, the supernatants were spun at 17,000 g for 15 min at 4°C. The 17,000 pellets were dissolved in Tris buffer and the protein concentration was measured with a BCA protein assay (Pierce, Rockford, IL). The Tris buffer was adjusted to 3% SDS, 8.7% glycerol, bromophenol blue, 30 mg/ml dithiothreitol, and pH 6.8 and heated 15 min at 65°C. Proteins were separated in 12.5% polyacrylamide gels (Criterion gels, Bio-Rad) at 100 V for 70 min and were electrotransferred onto Hybond-ECL nitrocellulose membranes (Amersham Biosciences) for 60 min at 100 V. Membranes were blocked in 5% nonfat dry milk in phosphate buffer consisting of 281 mM Na^+^, 100 mM Cl^−^, 21 mM H_2_PO_4_^−^, 80 mM HPO_4_^2−^, 0.1% Tween 20, and pH 7.5 for 1 h at room temperature and incubated overnight at 4°C with primary antibodies. The antibody-antigen reactions were visualized with enhanced chemiluminescence system (ECL Plus Western Blotting detection system, GE Lifesciences) and exposed to photographic film (Hyperfilm ECL, GE Lifesciences). Films were scanned on a flatbed scanner at 8-bit depth and 600 dpi resolution and bands semiquantified after background subtraction within a linear range using ImageJ software.

### Immunohistochemistry

Fixed kidneys were dehydrated in graded ethanol (70%, 96%, and 99%) for 2 hours each and left overnight in xylene. The tissue was embedded in paraffin wax, cut into 2 µm thick sections on a rotary microtome (Leica), and placed on Super Frost slides. Sections were dewaxed in xylene and rehydrated in graded ethanol. Endogenous peroxidase was blocked in 35% H_2_O_2_ in methanol. To retrieve antigens, sections were boiled in a microwave oven in TEG-buffer pH 9 with 10 mM Tris and 0.5 mM EGTA. Aldehydes were quenched in 50 mM NH_4_Cl in PBS, and the sections were blocked in 1% BSA, 0.2% gelatin, 0.05% Saponin in PBS. Then, sections were incubated with primary antibody diluted in 0.1% BSA, 0.3% Triton X-100 in PBS overnight at 4°C, and rinsed in 0.1% BSA, 0.2% gelatin, 0.05% Saponin in PBS.

For light microscopy, sections were incubated 1 hour with horseradish peroxidase conjugated secondary antibody in 0.1% BSA, 0.3% Triton X 100 in PBS and washed in 0.1% BSA, 0.2% gelatin, 0.05% saponin in PBS before visualization with diaminobenzidine in 35% H_2_O_2_ for 10 minutes. The sections were counterstained with Mayers hematoxylin and rinsed in running tap water before dehydration in graded ethanol and xylene and mounting with coverslips using Eukitt (CellPath). For immunofluorescence staining, the blocking of peroxidase was omitted and fluorophore-tagged secondary antibodies were applied. Where indicated, Topro3 (Invitrogen) was applied as a nuclear stain. Coverslips were mounted with a hydrophilic mounting media containing antifading reagent (glycergel, DAKO).

### Light microscopy and image processing

Brightfield imaging was performed on a Leica DMRE light microscope with PC APO 63x/1.32-0.6 NA and PC Fluotar 25x/0.75 NA oil immersion objectives, and a Leica DC 300 digital camera. Fluorescence imaging was performed on a Leica DM IRE2 inverted confocal microscope using a Leica TCS SP2 laser mole and an HCX PC APO CS 63x/1.32 NA oil objective. Images were acquired with 8-bit image depth, 1024×1024 pixel resolution, with an image averaging of 6 frames. For quantitation of staining intensities, laser power and settings for PMT gain and offset was kept constant for each antibody and adjusted to the brightest section. Image-Pro Analyzer and ImageJ were used for semi-quantitation and merging the confocal images. For semi-quantitation, the tubular outline was defined, then the cell area was determined, and the background-corrected fluorescence signal determined. The fluorescence signal, particle size or numbers were then normalized to the total tubule cell area (Figure S1). For calculation of colocalization, the Manders' coefficients were determined using Imaris 5.5 software (Bitplane) after thresholding the images. This is the optimal measure of colocalizing spot-like structures from two color channels on a dark background, as all pixels without signal above threshold are ignored (http://www.svi.nl/ColocalizationCoefficients).

### Immuno-gold electron microscopy

Approximately 1 mm^3^ renal cortical blocks were cut from the fixed male rat kidneys, infiltrated overnight in 0.01 M PBS with 2.3 M sucrose and 2% paraformaldehyde, mounted on holders, and rapidly frozen in liquid nitrogen. Tissue blocks with random orientation were cryosectioned with a Reichard FCS Reichert Ultracut S (Leica Microsystems, Wetzlar, Germany) at −120°C. The 80-nm cryosections were first blocked by incubation in PBS containing 0.05 M glycine and 0.1% skimmed milk powder. The sections were then incubated for 1 hour at room temperature with primary antibodies in PBS containing 0.1% skimmed milk powder. The primary antibodies were visualized using gold-conjugated secondary antibodies in PBS with 0.1% skimmed milk powder and polyethyleneglycol (5 mg/ml). For double labeling with same species primary antibodies, the antibodies were applied sequentially and with an intermittent blocking step after the first secondary antibody with rabbit serum and an excess of goat Fab fragments to avoid cross reactivity. Controls included single labeled sections and omission of either of the two primary antibodies. The cryosections were counter stained 10 minutes with 0.3% uranyl acetate in 1.8% methyl-cellulose and examined in a FEI Morgagni electron microscope.

### Immunoprecipitation

Immunoprecipitation was performed on kidney homogenate from aldosterone treated and control rats with the Santa Cruz H-95 Antibody (Table 1). First, the protein concentration in samples was measured using BCA protein assay reagent (Thermo Fisher Scientific). Then, volumes of kidney homogenate containing 350 µg protein were incubated with 40 µg of antibody in RIPA buffer containing 1% SDS, 0.05 M EDTA, 0.1 M Tris-HCl, 1% Na-dehydroxylate, 0.2 M NaCl, 1% Na Nonidet, and protease inhibitor. The mixture was incubated at 4°C for 1 hour. Subsequently, an equivalent volume of protein A beads were added to reaction mixtures and incubated with gentle rotation for 1 hour at room temperature. Then, samples were washed using RIPA buffer and eluted in sample buffer. Controls included omission of H-95 antibody and omission of beads. Samples were separated on 4–15% 26-well criterionTM TGXTM precast gel (BioRad), stained with Coomassie blue and lanes were excised manually before mass spectrometry.

### Liquid chromatography Tandem Mass Spectrometry (LC-MS/MS) analysis

LC-MS/MS was performed using a TripleTOF 5600 mass spectrometer (AB Sciex) operated under Analyst TF 1.5.1 control. The NanoSpray III source (AB Sciex) of the TripleTOF 5600 was connected in-line to an EASY-nLC II nano-HPLC system (Thermo Scientific). The HPLC was set-up to form a binary gradient of 0.1% formic (buffer A) acid and 90% acetonitrile (buffer B) at a flow rate of 250 nl/min. The trypsin digested samples were dissolved in 0.1% formic acid, injected, trapped and desalted isocratically on a ReproSil-Pur C18-AQ column (5 µm, 2 cm×100 µm I.D; Thermo Scientific) after which the peptides were eluted from the trap column and separated on an analytical ReproSil-Pur C18-AQ capillary column (3 µm, 10 cm×75 µm I.D.; Thermo Scientific) using a 50 min gradient from 5% buffer A to 35% buffer B. An Information dependent acquisition method was employed to automatically run experiments acquiring up to 50 MS/MS spectra per cycle using 2.8 sec cycle times and an exclusion window of 10 sec.

### Protein identification

The collected MS files were converted to Mascot generic format (MGF) using the AB SCIEX MS Data Converter beta 1.1 (AB SCIEX) and the "proteinpilot MGF" parameters. The peak lists were used to interrogate the Swiss-Prot (version 2012_04, 535,698 sequences) Rattus (7750 sequences) and Homo sapiens (20,250 sequences) databases using Mascot 2.3.02 (Matrix Science). Trypsin was employed and allowed one missed cleavage. Propionamide was chosen as a fixed modification and, oxidation of methionine was entered as a variable modification. The mass accuracy of the precursor and product ions were 10 ppm and 0.4 Da, respectively, and the instrument setting was specified as ESI-QUAD-TOF. The significance threshold (p) was set at 0.01 and with an ion score cut-off at 30. Mascot results were parsed using MS Data Miner v. 1.0 [18], protein hits were automatically validated if they satisfied one of the following criteria (i), identification based on one or more unique peptides with ion score above or equal to 45, or (ii), identification based on two or more unique peptides with ion score above or equal to 25. Spectra for protein hits only identified with one peptide within aldosterone treated rats were manually validated by assignment of significant peaks and occurrence of uninterrupted y- or b-ion series of at least 3 consecutive amino acid residues.

### Statistics

Data from semi-quantitative immunoblotting and immunofluorescence histochemistry were tested by two tailed t-tests choosing a significance level of p<0.05. Data in bar graphs are shown as mean ± SEM.

## Results

### Validation of aldosterone-induced αENaC abundance increase in the renal cortex

Aldosterone treatment significantly increased the abundance of a 90 kDa full-length band αENaC and a NH_2_-terminus cleaved fragment of 30 kDa (Figure S2A and S2B, p<0.001, n = 5). Immunostaining cryostat sections verified the aldosterone induced apical accumulation of αENaC subunits (Figure S2C, lower panel compared to top panel). Plasma aldosterone levels were 352.6±124.3 pmol/l in controls and 1190.2±210.2 pmol/l in aldosterone treated rats (n = 11, p = 0.003) similar to an identical experimental protocol [19], [20], adrenalectomized rats substituted with dexamethasone and aldosterone [21], and models of low Na^+^ diet [20], [22].

### 60 s ribosomal subunit L22 accumulate in distal tubules from aldosterone treated rats

**Figure 1:**
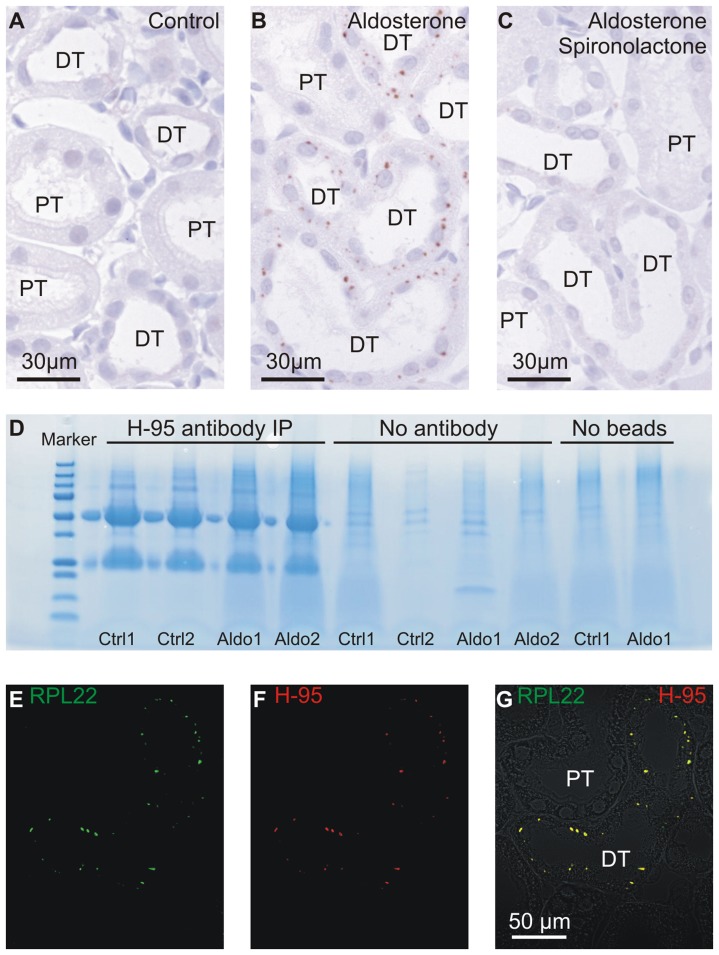
Aldosterone induces accumulation of punctate RPL22 immunoreactivity in distal renal tubules. A) Negative immunostaining of renal cortex from control rats (H-95, Table 1). B) Punctate immunolabeling of renal cortex from aldosterone treated rats with the same antibody. C) Sections from aldosterone plus spironolactone treated rats were stained in parallel to those shown in A and B. Kidney sections from aldosterone treated rats were double stained with H-95 and RPL22 antibodies. D) Coomassie blue stained gel showing the eluates from immuno-precipitation experiments with the H-95 antibody. Ctrl are controls, Aldo are aldosterone treated rat samples. E) Single channel signal from RPL22 staining. F) Single channel signal from H-95 staining. G) The merged channels (H-95 red, RPL22 green) overlaid on differential interference contrast image (DIC). “PT” marks proximal tubules, while “DT” indicates distal renal tubules and connecting tubules.

**Figure 2:**
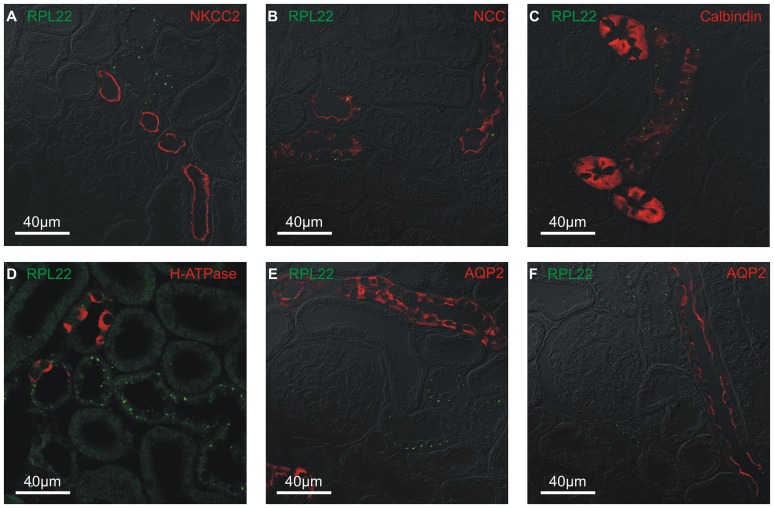
Identification of the tubular segment and cell structures displaying aldosterone-induced punctate immunoreactivity. Example of double immunofluorescence staining with RPL22 (green) and antibodies against tubule markers (all in red): NKCC2 (A), NCC (B), calbindin-D_28K_ (C), H^+^-ATPase (D), and AQP2 (E & F) in kidney cortex from aldosterone treated rats. Fluorescence signals are overlaid on the corresponding DIC image.

We sought to reproduce and validate previously unpublished observations of intracellular inclusions in renal tubules upon hormone treatment to further analyze the phenomenon. Antibodies directed against various transport proteins presumably bound accumulated protein in an unspecific manner, as they did not respect the segmental expression pattern of the intended target protein. Screening a panel of primary antibodies against renal Na^+^ transport proteins, we identified one (H-95, Table 1) that reproduced the inclusion-like punctate staining selectively in renal tubules in kidney sections from aldosterone treated rats (Fig. 1A and B). Similar results were obtained in three separate sets of aldosterone administration experiments (n = 5, 6, and 5, respectively). Co-administration of aldosterone and the mineralocorticoid receptor inhibitor spironolactone prevented formation of the immunoreactivity (Fig. 1C) [19]. The H-95 antibody was shown not to target its intended antigen, the sodium channel αENaC by immunoblotting (not shown) and a lack of apical immunolabeling of principal cells. Furthermore, H-95 immunoprecipitation of kidney homogenates from aldosterone treated and control rats (Figure 1D) followed by quantitative mass spectrometry identified three candidate proteins for H-95 binding: 60 s ribosomal subunit L22 (RPL22), 60 s ribosomal subunit L27, and moesin (Table 2). Distal tubules only displayed intracellular punctate immunoreactivity towards RPL22 (Fig. 1E). The identification of RPL22 as the H-95 immunoreactive protein was further verified by 1) identical labeling patterns in renal tubules upon aldosterone administration, when H-95 was applied first (Fig. 1E-G), 2) application of the RPL22 antibody prior to H-95 blocked the majority of H-95 labeling, indicating that the RPL22 antibody masked an epitope necessary for H-95 binding. Interestingly, RPL22 immunoreactivity developed gradually from 1 to 7 days of aldosterone administration (Figure S3). RPL22 punctae were not produced in rats treated with low-Na^+^ diet for 5 days reaching similar aldosterone levels as the aldosterone infusion (n = 5). Thus, the protein accumulation depends on excess aldosterone activating the MR on a background of normal plasma Na^+^.

**Table 2:** LC MS/MS analysis of proteins pulled down by H95 preferentially in samples from aldosterone treated rats but not detected in IP without beads or without antibody.

Protein		Aldo1	Aldo2	Ctrl1	Ctrl2
60S ribosomal protein L22	emPAI	0.23	0.23	n.d.	n.d.
	score	74	103		
60S ribosomal protein L27	emPAI	0.22	0.22	n.d.	n.d.
	score	29	48		
Moesin	emPAI	0.15	0.21	n.d.	n.d.
	score	127	145		

Ctrl: control, Aldo: aldosterone treated, n.d.: not detected.

### The aldosterone-induced RPL22 punctate are confined to DCT and CNT

RPL22 positive renal cortical tubules were identified by double immunofluorescence staining with the distal tubule markers: NKCC2 (TAL, Fig. 2A), NCC (DCT, Fig. 2B), calbindin-D_28k_ (DCT2, CNT, Fig. 2C), the intercalated cell marker H^+^-ATPase (DCT2 through CD, Fig. 2D), and principal cell marker AQP2 (CNT through CD; Fig. 2E and F, respectively). Aldosterone-induced immunoreactivity was mainly found in distal tubules with NCC expression and low/zero calbindin-D_28k_ staining (i.e. DCT1 and DCT2), with few punctae in tubular segments with high calbindin-D_28k_ abundance (CNT). Labeling was never observed in the H^+^-ATPase expressing cells (intercalated cells found in DCT2, CNT, CD, Fig. 2D). Immunostaining was mainly found in AQP2 negative distal tubule segments (DCT), with few punctae in AQP2 positive segments (DCT2 and CNT). No RPL22 labeling was observed corresponding to CD (Fig. 2F). No staining was observed in tubules with brush border, i.e. the proximal tubules. Taken together, these results indicate that the punctate immunoreactivity is confined mainly to DCT and less in CNT principal cells.

### Immunoreactive punctae co-localize with proteasome-containing aggregates

**Figure 3:**
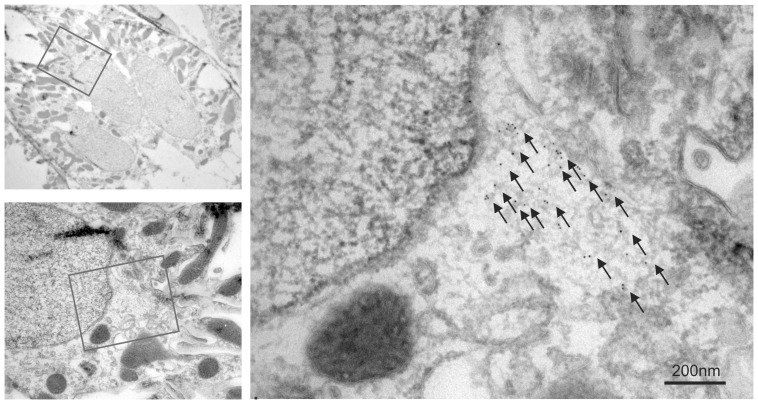
Immuno-gold electron micrographs of anti-RPL22 stained cryo-sections. Top left panel is a cellular overview of a distal renal tubule and the red box indicates the area magnified in left bottom panel. Here, the red box corresponds to the highest magnification electron micrograph (right panel). Arrows point to gold particles.

**Figure 4:**
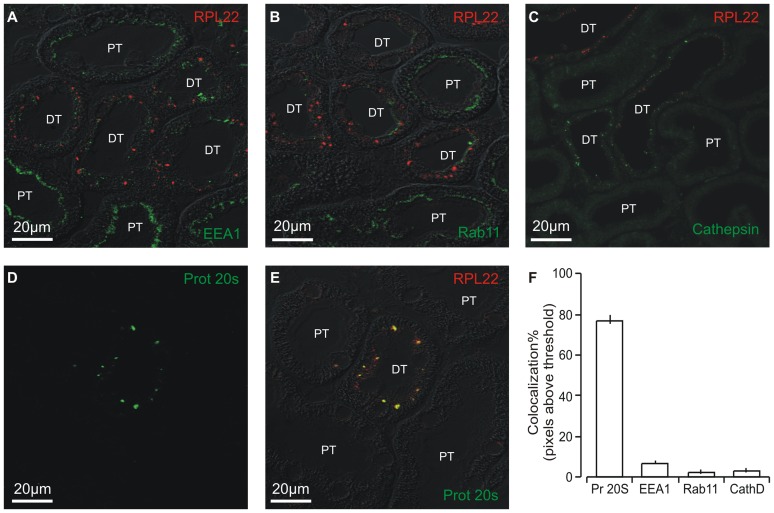
RPL22 colocalizes with aggregates containing the proteasome 20 s subunit. Double labeling immunofluorescence staining was performed on sections of paraffin embedded tissue from aldosterone treated animals for RPL22 and organelle markers from the endocytotic pathway or proteasome subunit. A) Co-labeling of the accumulated protein (red) and an early endosome marker, anti-EEA1 (green) overlaid on DIC image. B) Co-labeling with a recycling endosome marker, anti-Rab11 (green) with DIC overlay. C) Co-labeling with a lysosome marker, anti-cathepsin D (green). D) The immunostaining pattern of the marker anti-proteosome 20 s (green). E) Overlay with RPL22 fluorescence signals (red) and the corresponding DIC image. Yellow color indicates colocalization. F) Quantitation of co-localization of the accumulated immunoreactive protein with the vesicular markers (mean values from four images from five aldosterone treated animals).

**Figure 5:**
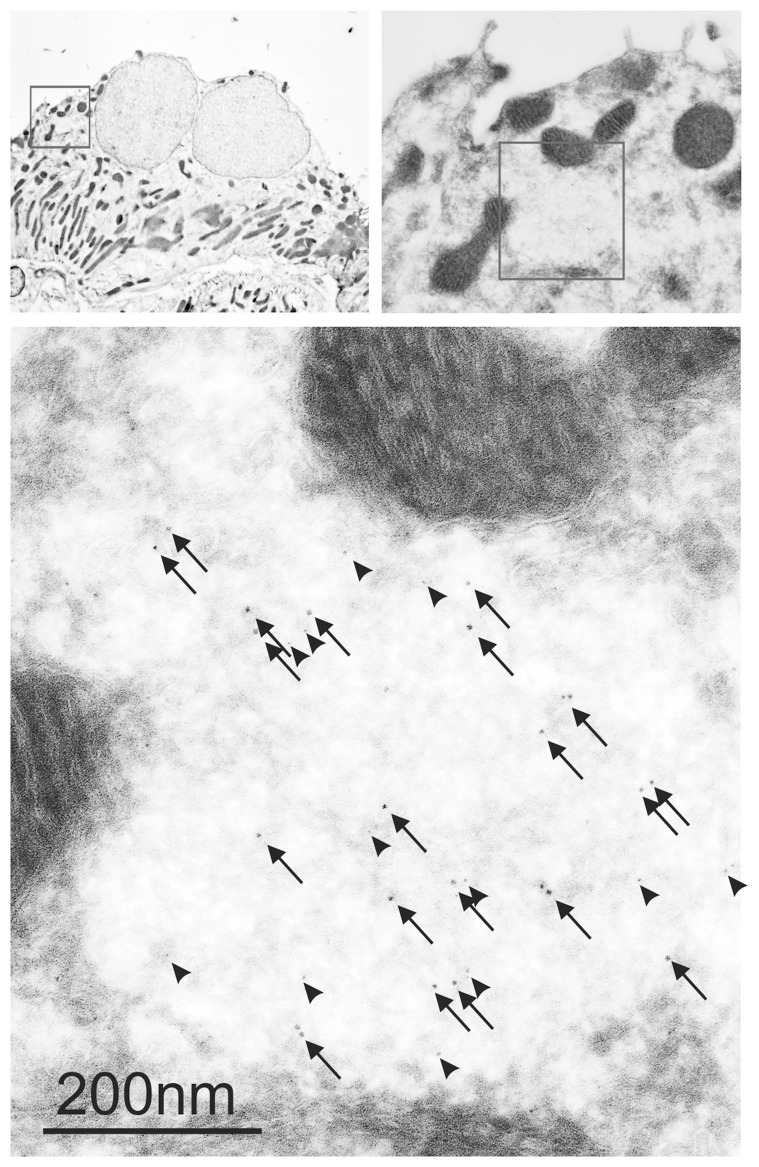
RPL22 colocalizes with the proteasome 20 s subunit by electron microscopy. Immuno-gold electron microscopy identifies the subcellular co-localization of RPL22 and proteasome subunit 20 s. Double labeling immuno-gold electron micrographs applying the H-95 antibody against aggregated RPL22 protein and proteasome 20 s on cryo-sectons. Top left panel is a cellular overview of a distal renal tubule and the red box indicates the area magnified in left bottom panel. The red box corresponds to the highest magnification electron micrograph bottom panel). Arrows point to 10 nm gold particles (proteasome 20 s) and arrowheads point to 5 nm gold particles (RPL22).

Electron microscopy revealed that the intracellular aggregates reside in distinct to small cytosolic areas without apparent relation to membrane structures (Fig. 3). Markers for various cellular structures were applied in an attempt to define the cellular site for the punctate RPL22 labeling. Minimal co-localization was observed with markers of early endosomes (EEA1, Fig. 4A), recycling endosomes (RAB11, Fig. 4B), or late endosomes/lysosomes (Cathepsin D, Fig. 4C). Figure 4D shows a punctate pattern of proteasome 20 s immunoreactivity in renal tubules from aldosterone treated rats. As single proteasomes form sub-resolution particles, the observed punctae most likely indicate a high degree of proteasome subunit aggregation. The proteasome staining pattern was virtually identical to the punctate RPL22 immunoreactivity (Fig. 4E). Analysis of similar images from 5 aldosterone treated rats showed 90–95% co-localization of the two signals. Thus, RPL22 punctate are almost exclusively restricted to aggregates containing proteasome 20 s subunits (Fig. 4F). Double-labeling electron microscopy with the proteasome 20 s and RPL22 antibodies showed clear colocalization in similar sized areas (Fig. 5), indicating that the punctae represent protein aggregates.

### Proteasome 20 s aggregation changes in renal tubules after aldosterone administration

**Figure 6:**
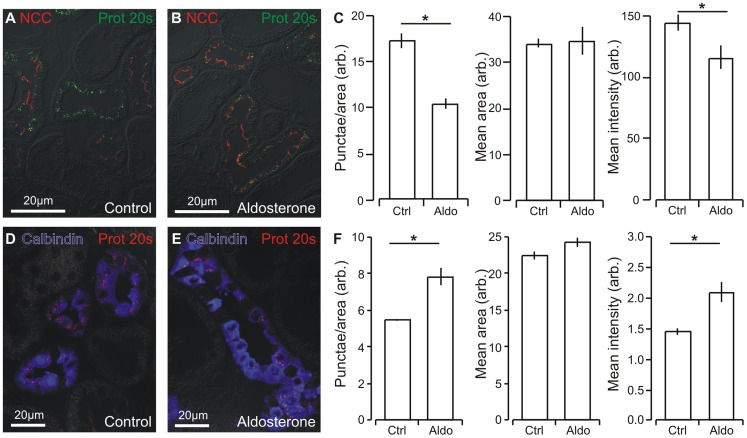
Aldosterone administration increases proteasome numbers and labeling intensity in distal renal tubules. A) Double labeling for proteasomes (proteasome 20 s, green) and a marker for DCT (NCC, red) in renal cortex from control rats. B) Similar fluorescence labeling in renal cortex from aldosterone treated rats. C) Quantitation of the mean number of proteasome-containing punctae, the mean area of these, and the mean proteasome 20 s immunoreactivity in the control and aldosterone treated groups in DCT (Con and Aldo, as indicated, n = 5). D) Double labeling of proteasomes (proteasome 20 s, red) and a marker for CNT (calbindin-D_28K_, blue) in renal cortex from control rats. E) Similar fluorescence labeling in renal cortex from aldosterone treated rats. F) Quantitation of the mean number of proteasome-containing punctae, the mean area of these, and the mean proteasome 20 s immunoreactivity in the control and aldosterone treated groups in CNT (Con and Aldo, as indicated, n = 5). * indicates statistical significance.

Punctate proteasome 20 s immunoreactivity was detectable only in distal renal tubules of rat kidneys as judged by the cellular co-localization with NCC for DCT (Fig. 6A) and calbindin-D_28K_ for CNT (Fig 6C). Proteasome 20 s staining intensity was weaker and less punctate in DCT from aldosterone treated rats as compared to control rats (Fig. 6A&B). Semi-quantitation confirmed that aldosterone treatment significantly decreased both the number of immunoreactive punctae per tubule area and the mean staining intensity per tubule area in DCT (Fig. 6C). By contrast, proteasome 20 s staining intensity was stronger and the punctate larger from aldosterone treated rats as compared to control rats in the CNT (Fig. 6D&E). Semi-quantitation confirmed that aldosterone treatment significantly increased both the number of immunoreactive punctae per tubule area and the mean staining intensity per tubule area in CNT (Fig. 6F). Thus, aldosterone administration seems to have opposing effect on proteasome aggregation in DCT and CNT.

### Renal tubular histone deacetylase 6 immunoreactivity in unaffected by aldosterone administration

**Figure 7:**
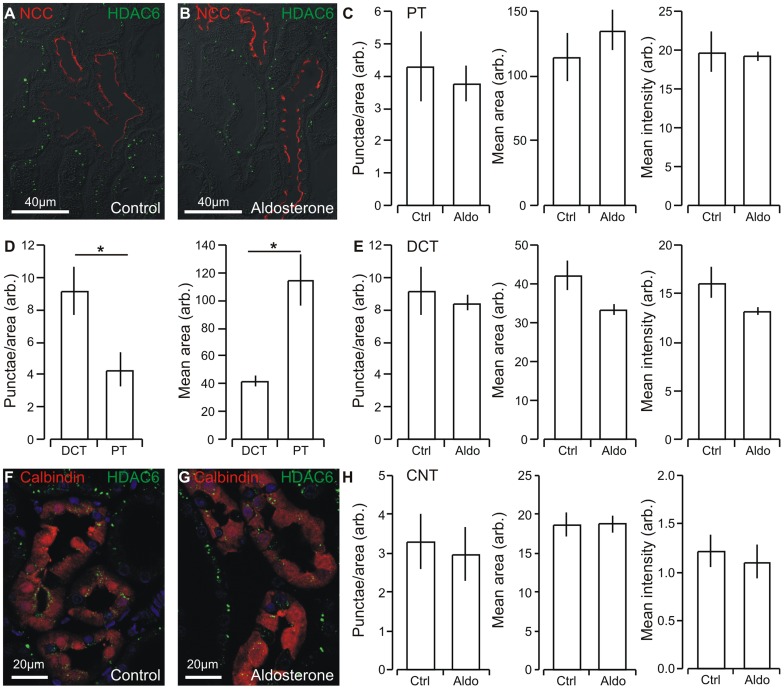
Aldosterone administration does not change HDAC6 staining in renal tubules. A) Double labeling for histone deacetylase 6 (HDAC6, green) and a marker for DCT (NCC, red) in renal cortex from control rats. Presence of brush border was used as selection criterion for PT. B) Similar fluorescence labeling in renal cortex from aldosterone treated rats. C) Quantitation of the mean number of HDAC6 punctae, the mean area of these, and the mean HDAC6 immunoreactivity in the control and aldosterone treated groups in PT (Con and Aldo, as indicated, n = 5). D) Comparizon of the mean number of HDAC6 punctae and the mean area of these in DCT and PT. E) Quantitation of the mean number of HDAC6 punctae, the mean area of these, and the mean HDAC6 immunoreactivity in the control and aldosterone treated groups in DCT (Con and Aldo, as indicated, n = 5).F) Double labeling histone deacetylase 6 (HDAC6, green) and a marker for CNT (calbindin-D_28K_, red) in renal cortex from control rats. G) Similar fluorescence labeling in renal cortex from aldosterone treated rats. H) Quantitation of the mean number of HDAC6 punctae, the mean area of these, and the mean HDAC6 immunoreactivity in the control and aldosterone treated groups in CNT (Con and Aldo, as indicated, n = 5). * indicates statistical significance.

Kidney sections were immunostained for the cytosol-to-aggresome cargo transfer protein histone deacetylase 6 (HDAC6). Punctate HDAC6 immunoreactivity was detectable in all renal cortical tubules renal tubules of rat kidneys as judged by the cellular co-localization with NCC for DCT (Fig. 7A) and calbindin-D_28K_ for CNT (Fig 7F) and the brush border appearance for proximal tubules (PT, Fig. 7A). HDAC6 puncta seemed unaffected from aldosterone treated rats as compared to control rats in PT and DCT (Fig. 7A&B). Semi-quantitation confirmed that aldosterone treatment did not affect the number of immunoreactive punctae per tubule area, the size, or and the mean staining intensity per tubule area in PT (Fig. 7C&E). The number of immunoreactive punctae per tubule area was significantly larger in DCT compared to PT, whereas the particle size was significantly larger in PT than DCT (Fig. 7D). HDAC6 staining intensity was unaffected by aldosterone treatment in the CNT (Fig. 7F&G). Semi-quantitation confirmed that aldosterone treatment had no significant effect on the number of immunoreactive punctae per tubule area and the mean staining intensity per tubule area in CNT (Fig. 7H). Thus, the renal tubular HDAC6 expression pattern is unaffected by aldosterone administration suggesting that aldosterone does not change the capacity to transfer ubiquitinated protein cargo from cytosolic protein aggregates to a putative aggresome.

### Aldosterone administration does not increase cargo transfer to aggresomes in distal renal tubules

**Figure 8:**
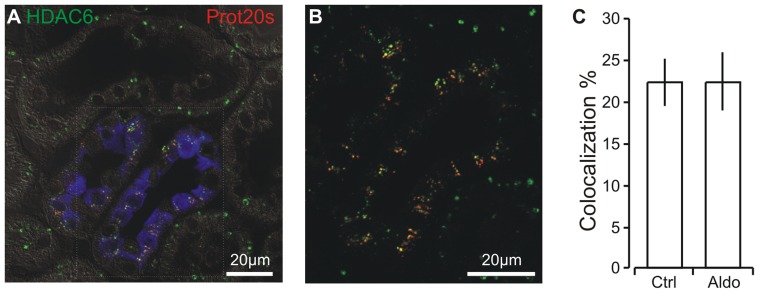
Analysis of the colocalization between proteasome 20 s aggregates and HDAC6. A) Triple fluorescence labeling for proteasome 20 s (red), aggresome (HDAC6, green), and calbindin-D_28K_ (blue) merged with the corresponding DIC image. B) Magnification of tubular structures in the same image. C) Quantitation of the co-localization of proteasome 20 s immunoreactive punctae and the aggresome transfer protein HDAC6 (n = 5, n.s.).

Kidney sections were immunostained for the cytosol-to-aggresome transfer protein histone deacetylase 6 (HDAC6) and proteasome 20 s. As illustrated in Figure 8A, HDAC6 and proteasome 20 s punctae were only coexpressed in distal tubules exemplified by the calbindin-D_28K_ positive CNT. In control conditions, the co-localization of the two proteins was modest (Fig. 8B). Aldosterone did not change the colocalization of HDAC6 and proteasome 20 s punctate (Fig. 8C). Thus, protein aggregates containing proteasome 20 s showed a modest degree of colocalization with the aggresome transfer protein HDAC6, and the aggregated proteasome subunits are not transported towards the autophagosome pathway even after aldosterone administration.

### Protein aggregation does not lead to aggresome formation in distal renal tubules

**Figure 9:**
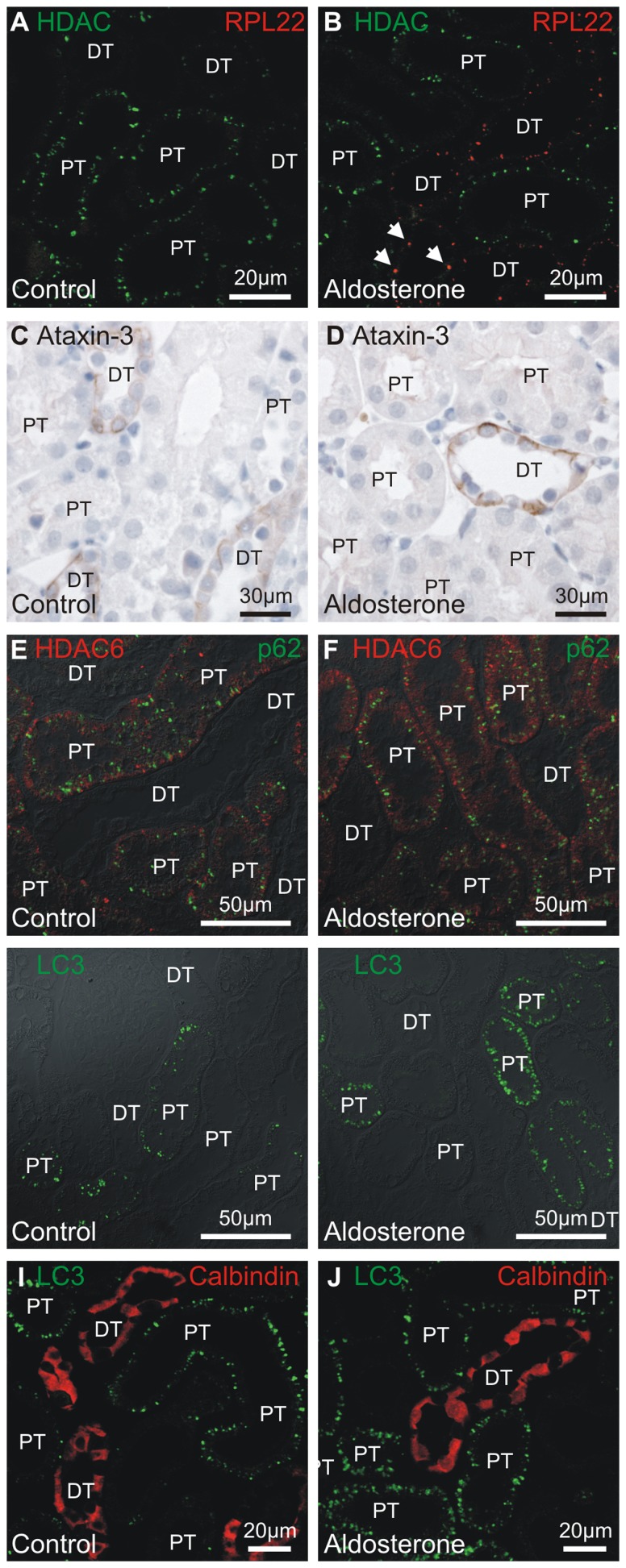
Co-localization of proteasome 20 s aggregates and HDAC6 after aldosterone administration. A) Double fluorescence labeling for RPL22 aggregates (H-95, red) and HDAC6 (green) in kidney from control rat. B) Similar labeling in kidney from aldosterone treated rat. Arrow heads indicate colocalization (in yellow). C) The immunostaing for the de-ubiquitinase ataxin-3 in control rat kidney sections. D) Representative micrograph of renal cortex of an aldosterone treated rat stained for ataxin-3. E) Double fluorescence labeling for aggresome initiator protein p62 (red) and HDAC6 (green) in kidney from control rat. F) Similar labeling in kidney from aldosterone treated rat. G) Renal cortex from control rats was labeled for autophagosomes by anti-LC3 antibodies (green) and overlaid on DIC image. H) Similar fluorescence labeling of renal cortex from aldosterone treated rat. I) Similar staining where distal renal tubules were identified by calbindin-D_28K_ (red). J) Corresponding micrograph from aldosterone treated rat. “PT” marks proximal tubules while “DT” are distal renal tubules and connecting tubules.

RPL22 and HDAC6 double labeling revealed only minimal co-localization between protein aggregates and the aggresome transfer protein HDAC6 (Fig. 9A and 9B). Transfer of protein cargo to aggresomes requires partial de-ubiquitination of the cargo by ataxin-3. Ataxin-3 immunoreactivity localized to the basal domain of the distal tubular cells in control rats (Fig. 9C), and aldosterone did not induce translocation of ataxin-3 to the protein aggregates (Fig. 9D). Similar staining patterns were obtained with a separate anti-ataxin3 antibody (not shown). The aggresome initiator protein p62 did not form distinct punctae in the distal tubules, but was present in most proximal tubule cells (Fig. 9E and 9F). Thus, classical aggresomes were absent from distal tubules as judged by the lack of immunoreactive p62 and larger HDAC6 punctae.

### Aldosterone treatment does not increase autophagosome formation in distal renal tubules

Autophagy is the only efficient cellular mechanism to clear aggresomes. Figures 9G and 9H show that autophagosomes, as assessed by LC3 immunoreactivity, pre-exist in renal proximal tubules. However, LC3 was absent from distal renal tubules from controls and from aldosterone treated animals (Fig. 9I and 9J, respectively). Autophagosomes were not observed in distal renal tubules by electron microscopical analysis even after aldosterone administration (n = 5).

### Aldosterone treatment induces lymphocyte infiltration and renal tubule vimentin expression

**Figure 10:**
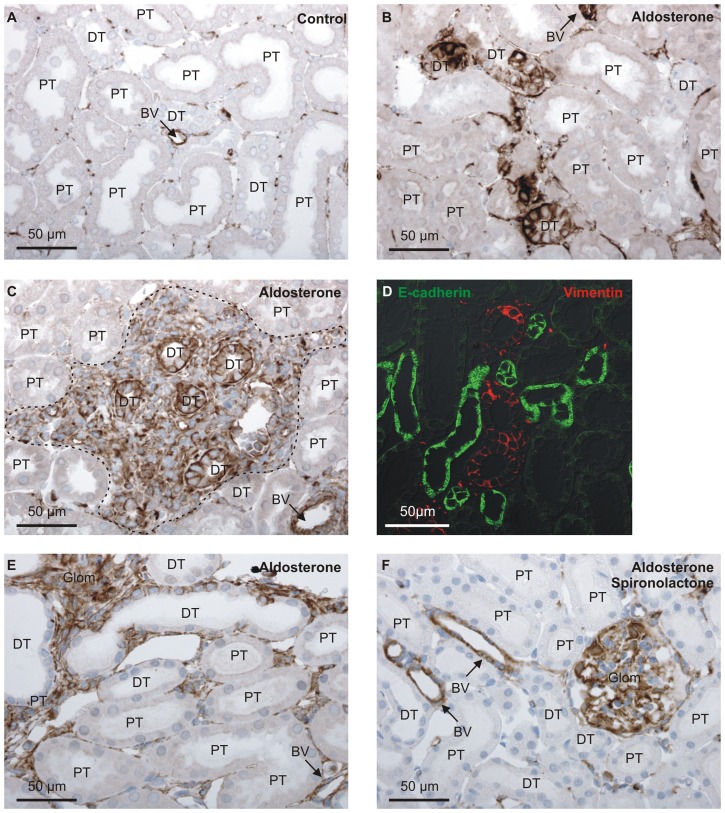
Distal renal tubular vimentin expression and cell infiltration. A) Typical vimentin immunoreactivity in control rat kidney. B) Similar staining of renal cortex from aldosterone treated rats. C) Lymphocyte infiltration was sometimes observed in the same areas where tubular vimentin expression was detected (dotted outline). D) Double fluorescence labeling for vimentin (red) and the cell-cell adhesion protein E-cadherin (green). E. Vimentin staining of a rat kidney cortex from aldosterone treated rat. F) Similar staining of a rat co-administered with aldosterone and spironolavctone from the same experiment. “Glom” are glomeruli, “BV” show blood vessels, “PT” marks proximal tubules, while “DT” indicates distal renal tubules and connecting tubules.

Vimentin is an intermediary filament specific to mesenchymal cells. In control kidneys, vimentin staining is confined to interstitial cells and perivascular cells (Fig. 10A), yet in aldosterone treated rats, a number of apparent distal tubular cells displayed marked vimentin immunoreactivity (Fig. 10B). Cell infiltration was found in the same areas in one of five rat kidneys (Fig. 10C). Although the vimentin positive tubules always resembled distal renal tubules morphologically by immunoperoxidase staining, a low brush border was frequently observed in these tubules by DIC. Furthermore, the low E-cadherin (Fig 10D) staining intensity as well as expression of the water channel AQP1 (not shown) of the vimentin positive tubules does not support a distal tubular affection. The number of vimentin positive tubules was quantified in a single midline section in each rat kidney from four independent experiments with 3, 5 or 6 rats in each group. Vimentin positive tubules were observed in 1 of 17 controls, 15 of 18 aldosterone treated rats, and 1 of 5 aldosterone and spironolactone co-treated rats. Figure 10 E exemplifies the renal tubular vimentin expression in an aldosterone treated rat from the experiment where one group of animas was co-administered aldosterone and spironolactone and did not display tubular vimentin staining (Fig. 10 F).

## Discussion

**Figure 11:**
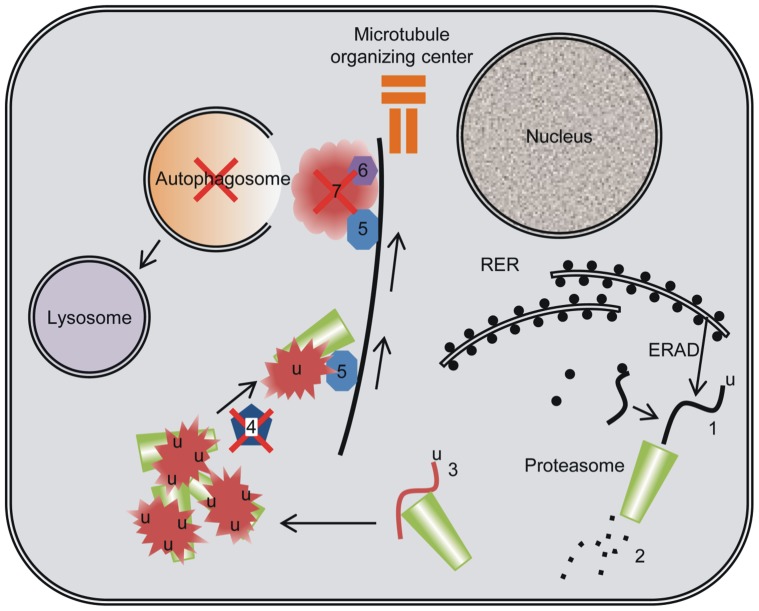
Model of the cellular protein degradation systems. After aldosterone treatment, distal renal tubular cells appear to have inadequate proteasomal capacity to degrade ubiquitinated protein (1) to short peptides (2). Instead, some cytosolic proteins, such as RPL22 (3) form aggregates with the proteasome subunits. In the absence of ataxin-3 (4), protein aggregates are not transferred by HDAC6 (5) to the aggresome (7) and aggregates can therefore not be cleared by autophagy.

The plasma aldosterone concentration can be elevated as a primary overproduction in the adrenal glands or as a response to high plasma K^+^ or angiotensin II levels. Among the clinically important causes for elevated aldosterone level, primary hyperaldosteronism is the most common cause of secondary arterial hypertension [23], and type 1 diabetes mellitus causes inappropriate activation of the entire renin-angiotensin-aldosterone axis [24]. In this study, we provide evidence that aldosterone levels elevated to a similar extent as the human conditions affect the ubiquitin-proteasome degradation system in distal renal tubule cells *in vivo*. Proteins seem to accumulate in aggregates containing 60 s ribosomal subunit L22 and proteasome 20 s subunits. The formation of aggregates, the increase in proteasome containing punctae, and the unchanged aggresomal transfer protein HDAC6 expression indicate that protein aggregates are not efficiently cleared by classical aggresome formation and autophagy in these tubules (Figure 11). This is in stark contrast to renal proximal tubules that express both aggresomes and autophagosomes even at resting conditions [25]. We also demonstrate that the same treatment induces epithelial cell expression of a mesenchymal cell marker and a case of massive cell infiltration; both possible consequences of the protein accumulation.

Cytosolic proteins, misfolded proteins and certain mature membrane proteins are degraded by the ubiquitin proteasome system [26]. As more protein is synthesized during aldosterone administration, a greater number of misfolded or unprocessed proteins from cytosolic production as well as from ER are expected to form inside the cells. Together with the general increased protein turnover, this increases the demand for efficient protein degradation. Thus, it is likely that prolonged aldosterone administration increases ERAD, UPR and as such the need for increased proteasomal activity in the aldosterone sensitive renal tubules. In the current study, aldosterone increased both the total proteasome 20S subunit abundance and the number of apparent aggregates containing proteasome subunits of a size around 1 µm in the CNT. This indicates that these renal tubular cells meet the higher demand for protein degradation by increasing protein breakdown capacity. The same data also represent the first indication that the proteasomes are unable to digest the complete load of proteins destined for degradation even in control distal renal tubules. This pattern was enhanced by aldosterone administration, upon which the proteasome 20S aggregates showed a high degree of co-localization with the cytosolic protein, RPL22. RPL22 displayed greater accumulation than the proteasome aggregates upon aldosterone administration, most likely exemplifying un-degraded protein cargo in the aggregates.

The formation of aggregates often induces partial de-ubiquitination of cargo proteins by ataxin-3 and microtubule-dependent transport of the entire aggregate to the aggresome. This process requires binding of the aggregate to the transfer protein HDAC6 [8], [9]. We did not observe punctate ataxin-3 expression in relation to the protein aggregates in the aldosterone sensitive renal tubules. This indicates that the partial de-ubiquitination preceding transfer of protein aggregates to aggresomes does not occur efficiently in these renal tubules. Furthermore, we found only a partial colocalization between the proteasome 20 s subunit and HDAC6. This reflects a defective transfer of protein aggregates to aggresomes after aldosterone administration, probably secondary to inefficient de-ubiquitination of the aggregated protein. In accordance with these suggestions, we did not find evidence for aggresome formation in distal renal tubules after aldosterone administration. In cell systems, the LC3 and ubiquitin binding protein p62 initiates aggresome formation [12], but this protein did not accumulate in distal renal tubules. This finding is striking considering the increased demand for efficient means of protein degradation during the hormone treatment.

Lysosomes normally clear aggregated proteins after incorporation of the aggresomes into autophagosomes [10]. We found no evidence for an efficient autophagosomal system in the distal renal tubules even after aldosterone administration. The autophagosome marker LC3 did label structures of the expected size in the renal proximal tubules. Despite the build-up of protein aggregates containing specific newly accumulated protein (such as RPL22) the distal renal tubules failed to develop classical aggresomes or autophagosomes. Thus, these tubules seem incapable of removing protein aggregates by autophagy upon aldosterone administration within the observation period applied. However, this distal renal tubules are not likely to be incapable in autophagy. On low dietary K^+^, rats develop hypokalemia and low plasma aldosterone. Under these conditions, animals displayed LC3-positive punctae specifically in distal renal tubules (personal communication with Sookkasem Khositseth and Trairak Pisitkun, Thailand). Thus, aldosterone or the changes in plasma K^+^ and/or Na^+^ may affect the distal tubular capacity for autophagy.

The cellular build-up of protein aggregates and/or aggresomes is a central feature of severe diseases of the central nervous system, liver and other epithelial tissues [11]–[13]. The apparent development of aggregates in renal epithelial cells suggests a potential role in aldosterone-dependent tubular damage with increased apoptosis and peritubular fibrosis. Such damage is observed after long-term elevated aldosterone and in diabetic kidney disease [27]–[29]. In support for a central influence of aldosterone in the pathogenesis, MR inhibition often ameliorates or even reverses severe kidney disease [29]–[32]. Our 7 days treatment did not lead to obvious apoptosis or severe fibrosis, perhaps because of the relatively short treatment period. Nevertheless, early signs of renal tubular dysfunction were observed in the form of focal distal tubular vimentin expression and in one case even cell infiltration. The epithelial cells in the same foci had non-detectable E-cadherin expression, which is a clear pathological sign of tubular stress.

In conclusion, we find that within 7 days of sustained elevated and clinically relevant concentrations of aldosterone, the distal renal tubular cells build up aggregates of un-degraded protein exemplified by RPL22. This aggregation occurs only in the aldosterone sensitive tubules and depends on MR activation. In contrast to proximal tubules, the distal tubular cells are remarkably unable to dispose of the aggregates through classical aggresome formation and autophagy upon aldosterone administration. This potentially damaging cellular protein aggregation leads to early focal tubular damage that also depends on MR activation. Short of autophagy-stimulating agents to aid protein aggregate removal in distal renal tubules, MR blockade may be the only means to ameliorate or prevent tubular cell stress and damage.

## Supporting Information

Figure S1 **Quantitative analysis of micrograph.** A) Segment-specific marker image (calbindin-D_28K_). B) Binary mask of analyzed tubules. C) The corresponding HDAC6 signal. D) Panel B was used to exclude HDAC6 signal from irrelevant areas. E) The particles were analysed after thresholding panel D, and normalized to the tubule cell area (panel B).
(TIF)

Figure S2 **Validation of aldosterone administration.** A) Immunoblotting of kidney cortex protein samples from control and aldosterone treated rats for αENaC (Loffing antibody). B) Densitometry for the full-length and NH_3_-terminal fragment (*: p<0.05, n = 5). C) αENaC immunofluorescence in kidneys from control (top panel) and aldosterone treated rat (bottom panel). Arrows: αENaC immunoreactivity.
(TIF)

Figure S3 **Time course of aldosterone induced protein accumulation.** Rats were treated with aldosterone for 0, 2, 4 or 7 days, as indicated. Two representative micrographs of the punctate RPL22 labeling are shown for each treatment period. “DT” marks distal renal tubules and cortical collecting ducts.
(TIF)
