# Supplementary figures and images for: Distal Renal Tubules Are Deficient in Aggresome Formation and Autophagy upon Aldosterone Administration
Source: PLoS One. 2014 Jul 7;9(7):e101258. doi: 10.1371/journal.pone.0101258 (PMC4085037; doi:10.1371/journal.pone.0101258)

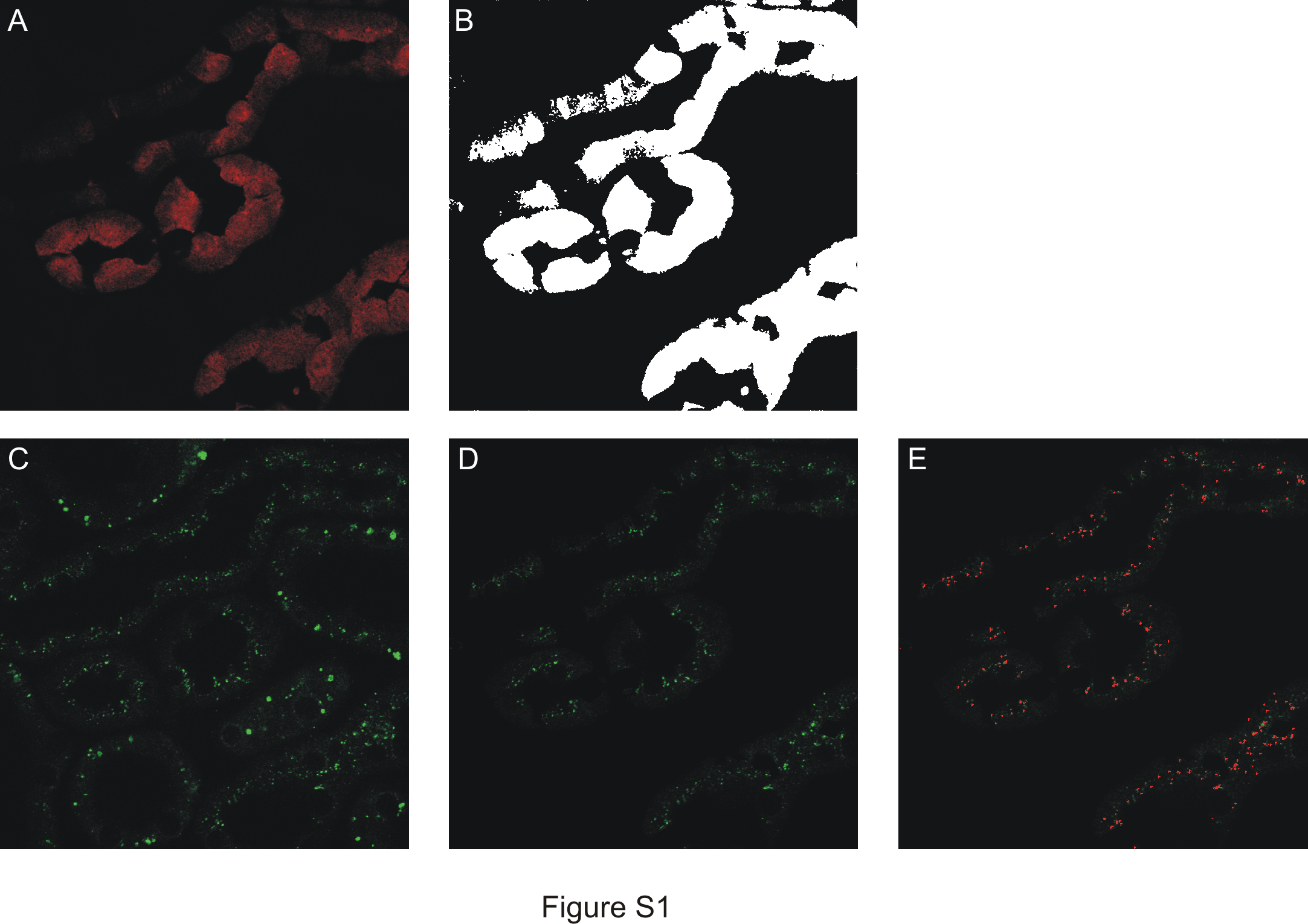

Supplement: Figure S1 — Quantitative analysis of micrograph. A) Segment-specific marker image (calbindin-D28K). B) Binary mask of analyzed tubules. C) The corresponding HDAC6 signal. D) Panel B was used to exclude HDAC6 signal from irrelevant areas. E) The particles were analysed after thresholding panel D, and normalized to the tubule cell area (panel B). (TIF) [file pone.0101258.s001.tif]

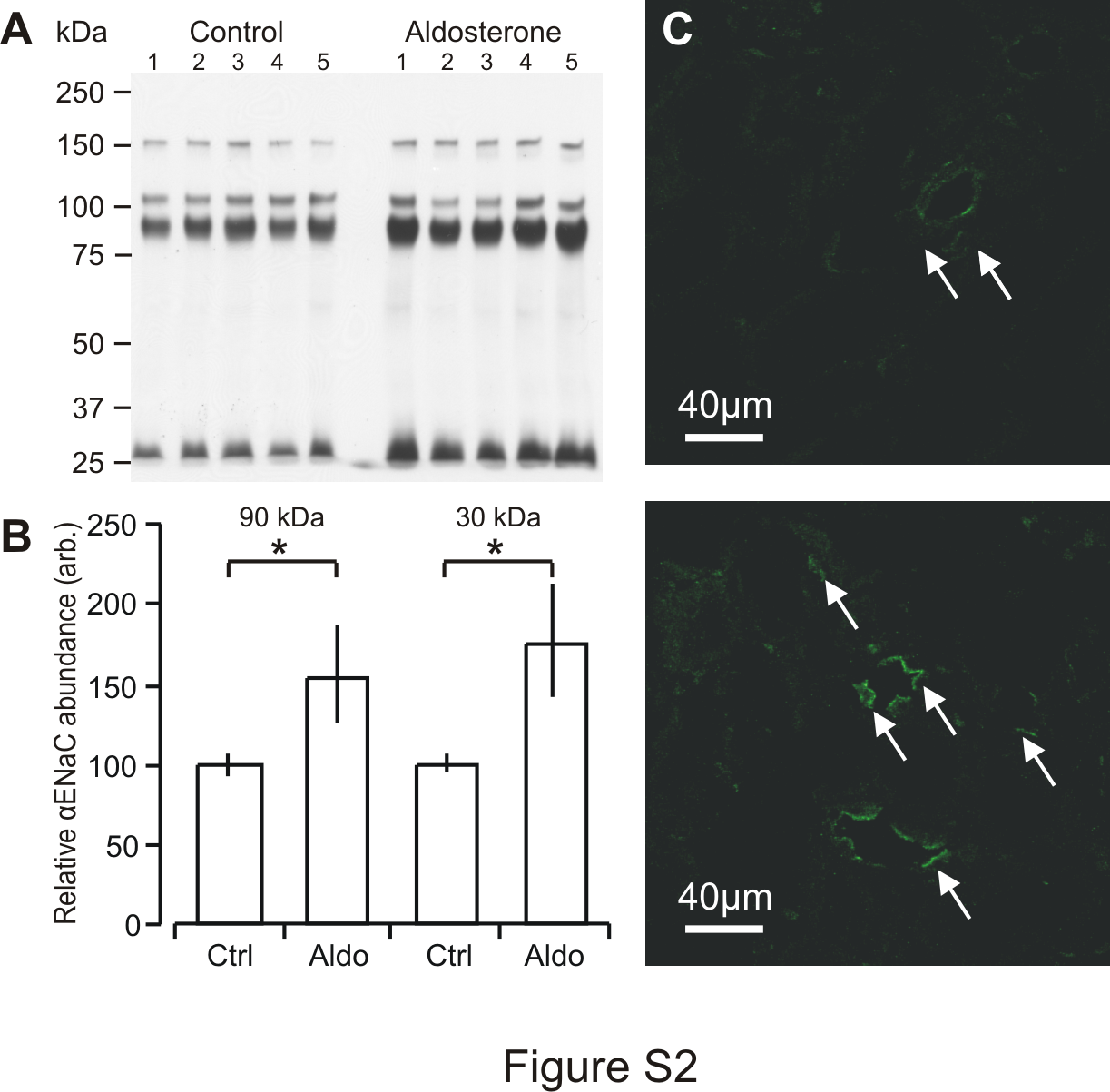

Supplement: Figure S2 — Validation of aldosterone administration. A) Immunoblotting of kidney cortex protein samples from control and aldosterone treated rats for αENaC (Loffing antibody). B) Densitometry for the full-length and NH3-terminal fragment (*: p<0.05, n = 5). C) αENaC immunofluorescence in kidneys from control (top panel) and aldosterone treated rat (bottom panel). Arrows: αENaC immunoreactivity. (TIF) [file pone.0101258.s002.tif]

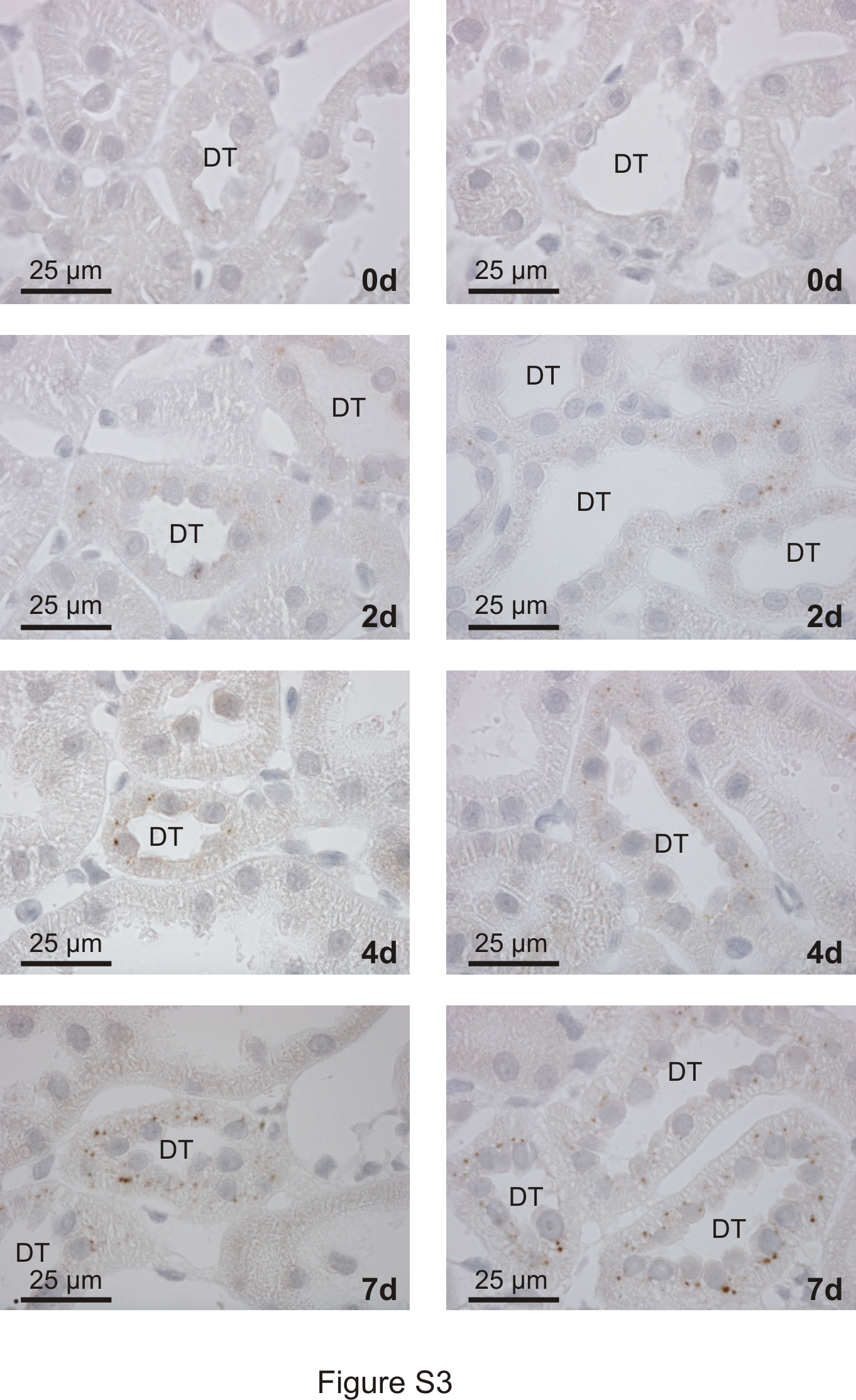

Supplement: Figure S3 — Time course of aldosterone induced protein accumulation. Rats were treated with aldosterone for 0, 2, 4 or 7 days, as indicated. Two representative micrographs of the punctate RPL22 labeling are shown for each treatment period. “DT” marks distal renal tubules and cortical collecting ducts. (TIF) [file pone.0101258.s003.tif]
